# Supplementary material for: Modeling glioblastoma heterogeneity as a dynamic network of cell states
Source: Mol Syst Biol. 2021 Sep 16;17(9):e10105. doi: 10.15252/msb.202010105 (PMC8444284; doi:10.15252/msb.202010105)
Supplement: Supplementary file 5 — Source Data for Figure 3 [file MSB-17-e10105-s001.zip › Figure3A_sourcedata/GSEA_3065/hallmarks_state2.GseaPreranked.1623416269049/HALLMARK_PROTEIN_SECRETION.html]

Details for gene set HALLMARK\_PROTEIN\_SECRETION[GSEA]

|  || Dataset | state2 |
| Phenotype | NoPhenotypeAvailable |
| Upregulated in class | na\_neg |
| GeneSet | HALLMARK\_PROTEIN\_SECRETION |
| Enrichment Score (ES) | -0.42479274 |
| Normalized Enrichment Score (NES) | -1.6330193 |
| Nominal p-value | 0.0040650405 |
| FDR q-value | 0.009733429 |
| FWER p-Value | 0.062 |
Table: GSEA Results Summary

  

Fig 1: Enrichment plot: HALLMARK\_PROTEIN\_SECRETION      
 Profile of the Running ES Score & Positions of GeneSet Members on the Rank Ordered List

  

| PROBE | GENE SYMBOL | GENE\_TITLE | RANK IN GENE LIST | RANK METRIC SCORE | RUNNING ES | CORE ENRICHMENT || 1 | ANP32E |  |  | 136 | 0.330 | 0.0297 | No |
| 2 | AP2S1 |  |  | 385 | 0.208 | 0.0310 | No |
| 3 | CLTA |  |  | 537 | 0.175 | 0.0382 | No |
| 4 | RAB2A |  |  | 672 | 0.153 | 0.0444 | No |
| 5 | GNAS |  |  | 744 | 0.145 | 0.0562 | No |
| 6 | COPE |  |  | 1016 | 0.118 | 0.0431 | No |
| 7 | SOD1 |  |  | 1028 | 0.117 | 0.0576 | No |
| 8 | ARF1 |  |  | 1068 | 0.114 | 0.0686 | No |
| 9 | TPD52 |  |  | 1493 | 0.087 | 0.0349 | No |
| 10 | TSG101 |  |  | 1925 | 0.067 | -0.0022 | No |
| 11 | ARCN1 |  |  | 1983 | 0.065 | 0.0004 | No |
| 12 | ZW10 |  |  | 1994 | 0.065 | 0.0080 | No |
| 13 | NAPA |  |  | 2063 | 0.062 | 0.0091 | No |
| 14 | AP3S1 |  |  | 2167 | 0.059 | 0.0059 | No |
| 15 | BNIP3 |  |  | 2196 | 0.058 | 0.0106 | No |
| 16 | SNX2 |  |  | 2206 | 0.057 | 0.0173 | No |
| 17 | ARFGAP3 |  |  | 2237 | 0.056 | 0.0216 | No |
| 18 | AP2M1 |  |  | 2310 | 0.053 | 0.0210 | No |
| 19 | AP3B1 |  |  | 2497 | 0.048 | 0.0075 | No |
| 20 | MAPK1 |  |  | 2595 | 0.045 | 0.0031 | No |
| 21 | BET1 |  |  | 2655 | 0.043 | 0.0025 | No |
| 22 | RPS6KA3 |  |  | 2677 | 0.042 | 0.0059 | No |
| 23 | VAMP7 |  |  | 2990 | 0.034 | -0.0229 | No |
| 24 | DNM1L |  |  | 3024 | 0.033 | -0.0219 | No |
| 25 | VAMP3 |  |  | 3171 | 0.030 | -0.0335 | No |
| 26 | RAB14 |  |  | 3306 | 0.027 | -0.0441 | No |
| 27 | RAB9A |  |  | 3375 | 0.026 | -0.0479 | No |
| 28 | SCRN1 |  |  | 3513 | 0.024 | -0.0594 | No |
| 29 | VPS4B |  |  | 3659 | 0.021 | -0.0721 | No |
| 30 | STAM |  |  | 3689 | 0.021 | -0.0724 | No |
| 31 | NAPG |  |  | 4016 | 0.015 | -0.1052 | No |
| 32 | AP1G1 |  |  | 4055 | 0.015 | -0.1073 | No |
| 33 | ARFIP1 |  |  | 4061 | 0.015 | -0.1059 | No |
| 34 | COG2 |  |  | 4098 | 0.014 | -0.1079 | No |
| 35 | OCRL |  |  | 4224 | 0.012 | -0.1197 | No |
| 36 | ICA1 |  |  | 4373 | 0.009 | -0.1343 | No |
| 37 | GLA |  |  | 4388 | 0.009 | -0.1346 | No |
| 38 | YKT6 |  |  | 4828 | 0.002 | -0.1812 | No |
| 39 | RAB22A |  |  | 4844 | 0.002 | -0.1826 | No |
| 40 | VAMP4 |  |  | 4961 | 0.000 | -0.1949 | No |
| 41 | TOM1L1 |  |  | 5013 | -0.000 | -0.2003 | No |
| 42 | SSPN |  |  | 5862 | -0.014 | -0.2891 | No |
| 43 | GBF1 |  |  | 5969 | -0.016 | -0.2983 | No |
| 44 | STX7 |  |  | 6062 | -0.018 | -0.3058 | No |
| 45 | GOSR2 |  |  | 6198 | -0.020 | -0.3176 | No |
| 46 | ARFGEF2 |  |  | 6299 | -0.022 | -0.3254 | No |
| 47 | SNAP23 |  |  | 6326 | -0.022 | -0.3252 | No |
| 48 | ATP7A |  |  | 6343 | -0.022 | -0.3239 | No |
| 49 | SEC22B |  |  | 6569 | -0.027 | -0.3444 | No |
| 50 | VPS45 |  |  | 6713 | -0.030 | -0.3557 | No |
| 51 | COPB2 |  |  | 6786 | -0.031 | -0.3592 | No |
| 52 | TMX1 |  |  | 6847 | -0.033 | -0.3613 | No |
| 53 | STX12 |  |  | 6986 | -0.036 | -0.3712 | No |
| 54 | SCAMP3 |  |  | 7093 | -0.039 | -0.3773 | No |
| 55 | KIF1B |  |  | 7160 | -0.041 | -0.3789 | No |
| 56 | RAB5A |  |  | 7229 | -0.043 | -0.3805 | No |
| 57 | MON2 |  |  | 7363 | -0.046 | -0.3885 | No |
| 58 | TMED2 |  |  | 7420 | -0.048 | -0.3881 | No |
| 59 | AP2B1 |  |  | 7436 | -0.048 | -0.3833 | No |
| 60 | STX16 |  |  | 7727 | -0.057 | -0.4066 | No |
| 61 | GOLGA4 |  |  | 7898 | -0.063 | -0.4164 | Yes |
| 62 | CAV2 |  |  | 7907 | -0.063 | -0.4088 | Yes |
| 63 | RER1 |  |  | 7968 | -0.066 | -0.4064 | Yes |
| 64 | SCAMP1 |  |  | 7974 | -0.066 | -0.3980 | Yes |
| 65 | ATP6V1H |  |  | 8025 | -0.068 | -0.3943 | Yes |
| 66 | USO1 |  |  | 8086 | -0.071 | -0.3912 | Yes |
| 67 | COPB1 |  |  | 8093 | -0.071 | -0.3824 | Yes |
| 68 | EGFR |  |  | 8094 | -0.071 | -0.3729 | Yes |
| 69 | SEC24D |  |  | 8096 | -0.071 | -0.3634 | Yes |
| 70 | M6PR |  |  | 8183 | -0.076 | -0.3624 | Yes |
| 71 | ERGIC3 |  |  | 8316 | -0.084 | -0.3653 | Yes |
| 72 | ARFGEF1 |  |  | 8352 | -0.086 | -0.3576 | Yes |
| 73 | PPT1 |  |  | 8369 | -0.087 | -0.3477 | Yes |
| 74 | CLN5 |  |  | 8453 | -0.093 | -0.3441 | Yes |
| 75 | CLTC |  |  | 8571 | -0.101 | -0.3431 | Yes |
| 76 | ABCA1 |  |  | 8635 | -0.106 | -0.3357 | Yes |
| 77 | SEC31A |  |  | 8793 | -0.122 | -0.3362 | Yes |
| 78 | ADAM10 |  |  | 8868 | -0.132 | -0.3264 | Yes |
| 79 | YIPF6 |  |  | 9078 | -0.162 | -0.3271 | Yes |
| 80 | IGF2R |  |  | 9107 | -0.167 | -0.3077 | Yes |
| 81 | ATP1A1 |  |  | 9121 | -0.170 | -0.2863 | Yes |
| 82 | CLCN3 |  |  | 9179 | -0.185 | -0.2677 | Yes |
| 83 | TMED10 |  |  | 9196 | -0.189 | -0.2442 | Yes |
| 84 | LMAN1 |  |  | 9216 | -0.199 | -0.2195 | Yes |
| 85 | PAM |  |  | 9280 | -0.224 | -0.1963 | Yes |
| 86 | LAMP2 |  |  | 9380 | -0.322 | -0.1638 | Yes |
| 87 | CD63 |  |  | 9394 | -0.353 | -0.1179 | Yes |
| 88 | CTSC |  |  | 9413 | -0.431 | -0.0621 | Yes |
| 89 | DST |  |  | 9422 | -0.485 | 0.0019 | Yes |
Table: GSEA details [plain text format]

  

Fig 2: HALLMARK\_PROTEIN\_SECRETION: Random ES distribution      
 Gene set null distribution of ES for **HALLMARK\_PROTEIN\_SECRETION**
